# Supplementary material for: Increased mRNA translation delays tumour initiation and exposes a therapeutic vulnerability in lung cancer
Source: Mol Cancer. 2026 May 21;25:172. doi: 10.1186/s12943-026-02680-z (PMC13366719; doi:10.1186/s12943-026-02680-z)

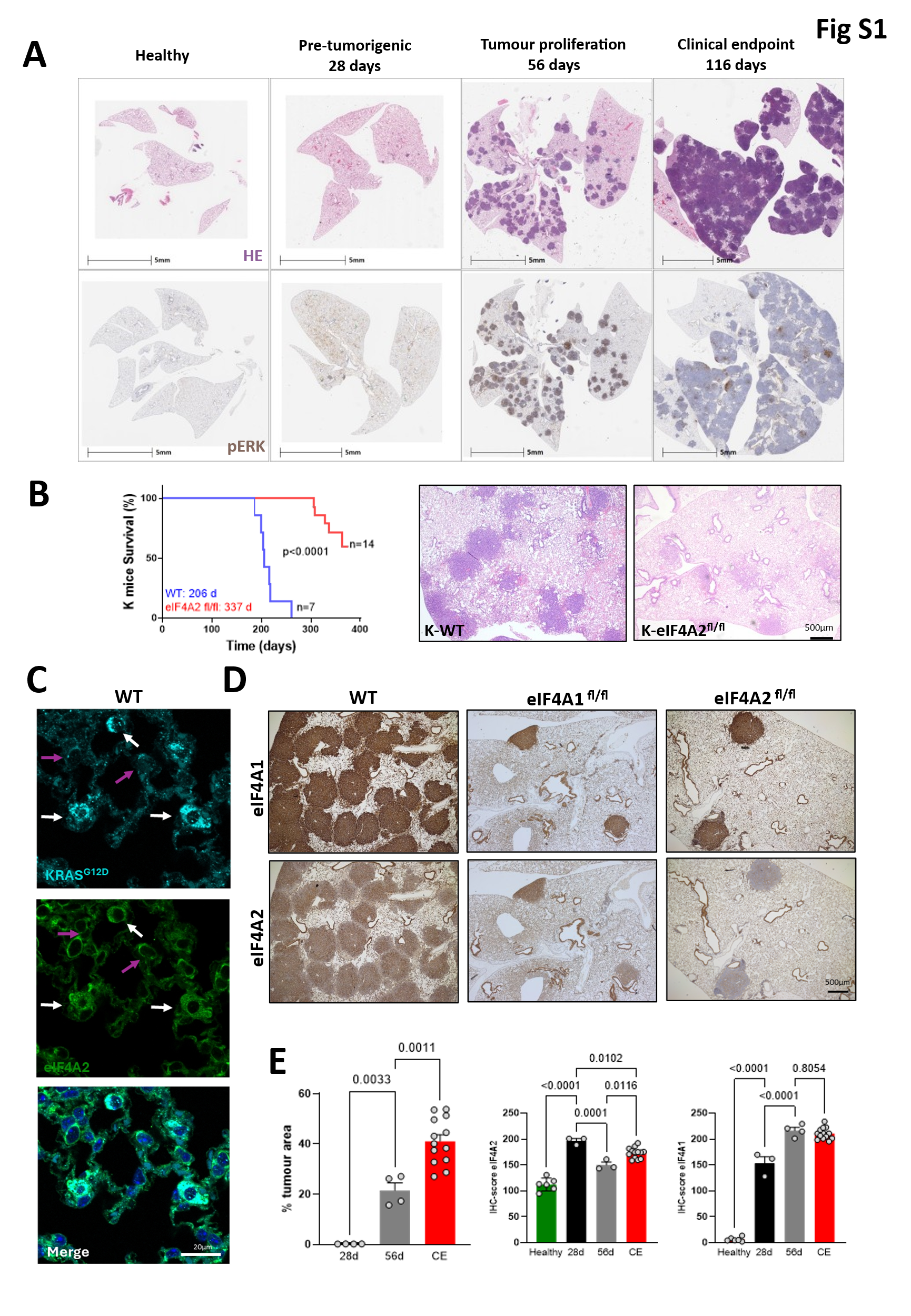


**Supplementary figure S1. KRAS-driven mouse of lung cancer and influence of eIF4A paralogues.**

(**A**) Ad5-SPC-CRE was administered intranasally to *Kras*^LSL-G12D/WT^; *Rosa26*^LSL-MYC/LSL-MYC^ (KM) mice at a titre (1 X 10^8^ plaque forming units (pfu)/mouse) sufficient to evoke recombination in ≈5% alveolar type II cells. Some mice were left uninduced (healthy). Mice were sacrificed at the indicated time points and lungs removed and fixed. Tumour burden was visualised using H&E and phospho-ERK by immunohistochemistry.

(**B**) *Kras*^LSL-G12D/WT^; *Eif4a2*^WT/WT^ or *Kras*^LSL-G12D/WT^; *Eif4a2*^fl/fl^ mice were induced with Ad5-SPC-CRE as for (A). Mice were sacrificed at clinical endpoint or, in the case of several animals from the *Kras*^LSL-G12D/WT^; *Eif4a2*^fl/fl^ cohort at 375 days following induction with Ad5-SPC-CRE. The H&E images display the tumour burden of a *Kras*^LSL-G12D/WT^; *Eif4a2*^WT/WT^ mouse at clinical endpoint (left panel) and the lung field of a *Kras*^LSL-G12D/WT^; *Eif4a2*^fl/fl^ mouse taken 375 days following Ad5-SPC-CRE administration that had not displayed clinical signs of lung cancer. The statistical test for the Kaplan-Maier analysis in is Logrank (Mantel-Cox) and cohort sizes were N=7 individual mice for *Eif4a2*^WT/WT^ (K-WT) and N=14 for K-*Eif4a2*^fl/fl^. Only 9 of the 14 mice in the *Eif4a2*^fl/fl^ cohort had reached clinical endpoint by 375 days following Ad5-SPC-CRE administration when the experiment was terminated.

(**C**) Ad5-SPC-CRE was administered to KM mice. Mice were sacrificed 28 days following and mutant KRASG12D (blue) and eIF4A2 (green) were visualised in lung slices using immunofluorescence. eIF4A2 expression is increased in KRAS^G12D^ positive cells (white arrows) and reduced in KRAS^G12D^ negative cells (magenta arrows).

(D) KM mice that were either eIF4A1/2^WT/WT^, eIF4A1^fl/fl^ or eIF4A2^fl/fl^ were induced with Ad5-SPC-CRE. Mice were sacrificed 56 days following Ad5-SPC-CRE administration and eIF4A1 and eIF4A2 visualised in the lung using immunohistochemistry.

(E) Quantification of the tumour area by H&E staining, and of eIF4A1 and eIF4A2 expression by IHC at different stages of tumour progression in the KM mice. Bars are mean ± SEM of N=3-14 individual mice. Statistical test is one-way ANOVA. (28d and 56d = days post Ad5-SPC-CRE administration, CE= clinical endpoint).


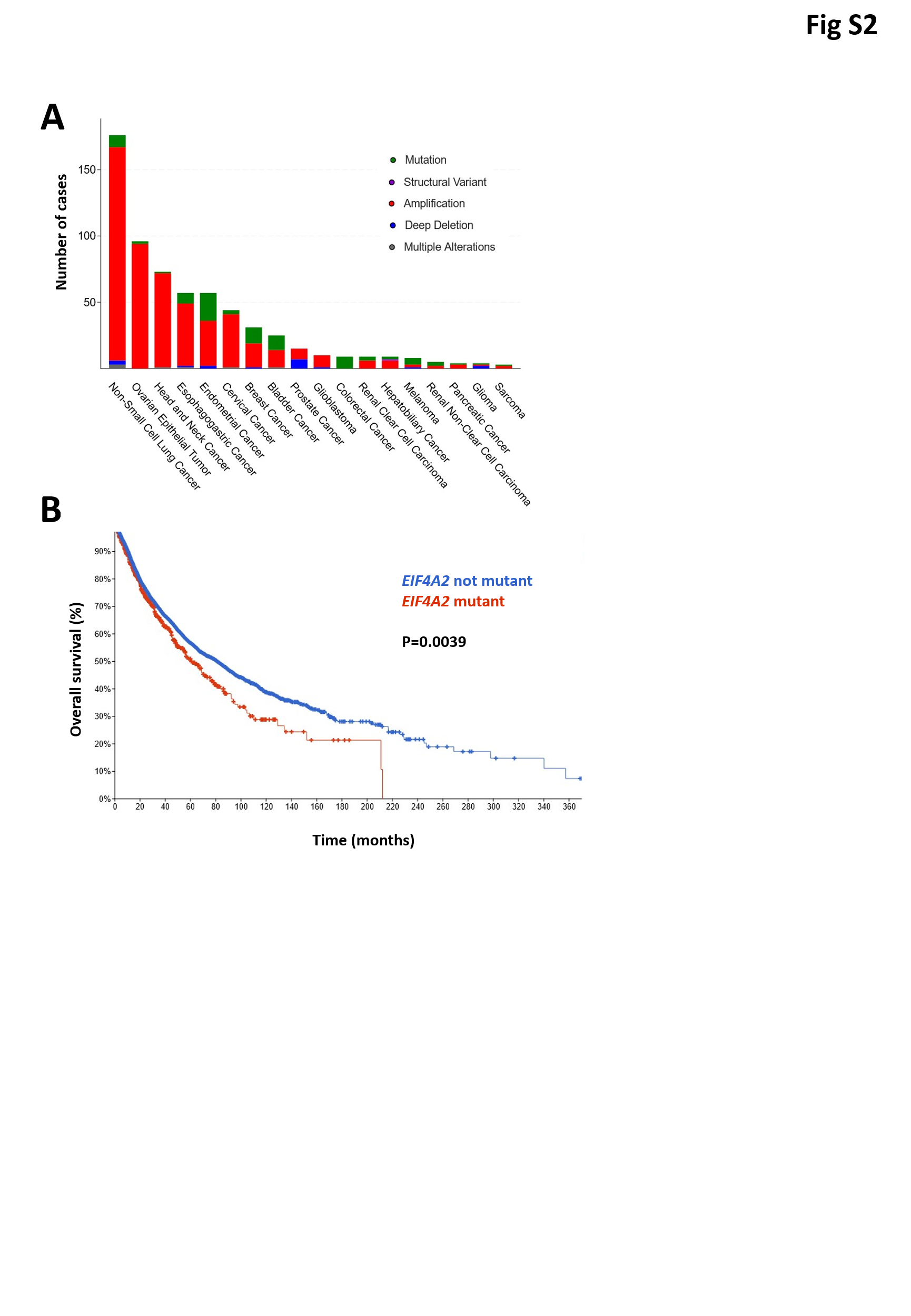


**Supplementary figure S2. Distribution of *EIF4A2* mutations across cancer types.**

(**A**) Number of patients with *EIF4A2* mutations were obtained from cBioportal by querying the TCGA dataset (10953 patients) [14]. *EIF4A2* mutations are present in 6% of cancers on average, but in 16.7% of Non-Small-Cell Lung Cancer. The majority of *EIF4A2* mutations in NSCLC lead to amplification of the gene.

(B) The TCGA dataset was used to interrogate the overall survival of patients with NSCLC which do (red, n=642) and do not (blue, n=10318) display mutation of the *EIF4A2* gene. Patients with NSCLCs harbouring mutations in *EIF4A2* exhibit poorer overall survival. The statistical test for the Kaplan-Maier analysis in is Logrank (Mantel-Cox).


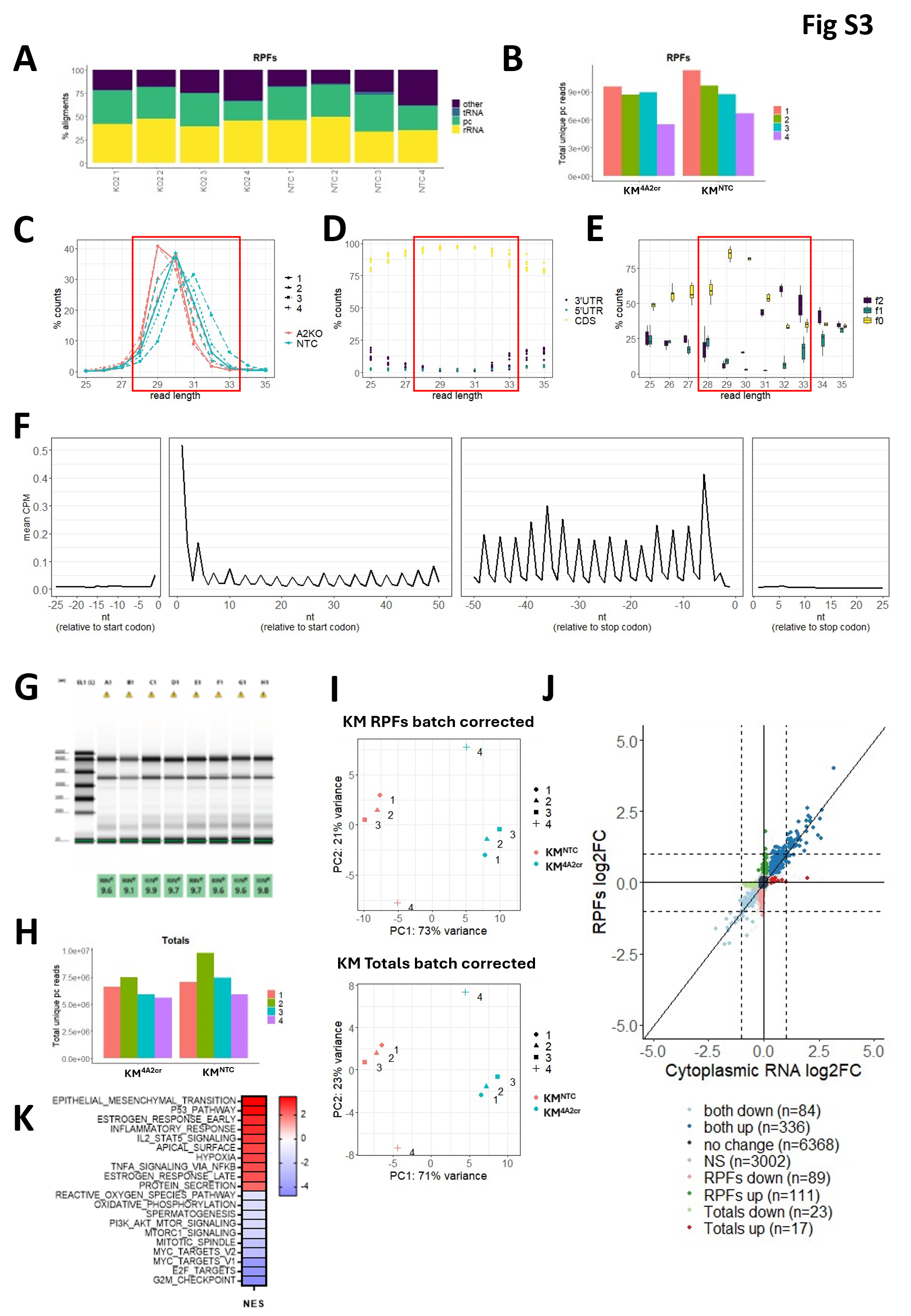


**Supplementary figure S3. RiboSeq Quality Control.**

(**A**) Alignment percentages across all ribosome-protected fragment (RPF) samples. (**B**) Total number of unique protein-coding reads for all RPF samples. (**C**) Distribution of read lengths for all protein-coding RPF samples. (**D**) Proportion of reads aligning to the 5'UTR, CDS, or 3'UTR for various read lengths. (**E**) Boxplot showing the percentage of RPF reads within each coding sequence frame, categorised by read length. The red boxes in panels C-E highlight the read lengths selected for further analysis. (**F**) Mean counts per million (CPM) for all transcripts and samples at the indicated positions, after applying the offsets described in the methods section, demonstrating periodicity specifically within the CDS. (**G**) Tapestation file indicating that total cytoplasmic RNA remains intact, with high RNA Integrity Number (RIN) values. (**H**) Total count of unique protein-coding reads from the total cytoplasmic RNA samples. (**I**) Batch corrected PCA plots showing distribution of the replicates per condition in RPF and total RNA. (**J**) KM^4A2cr^/KM^NTC^Log2FC of RPFs vs total RNA showing increased translational efficiency in KM^4A2cr^. (**K**) Gene Set Enrichment Analysis of KM^4A2cr^/KM^NTC^ Log2FC total RNA showing significantly enriched (red) or depleted (blue) hallmarks in KM^4A2cr^ cells ranked by the Normalised Enrichment Score (NES).


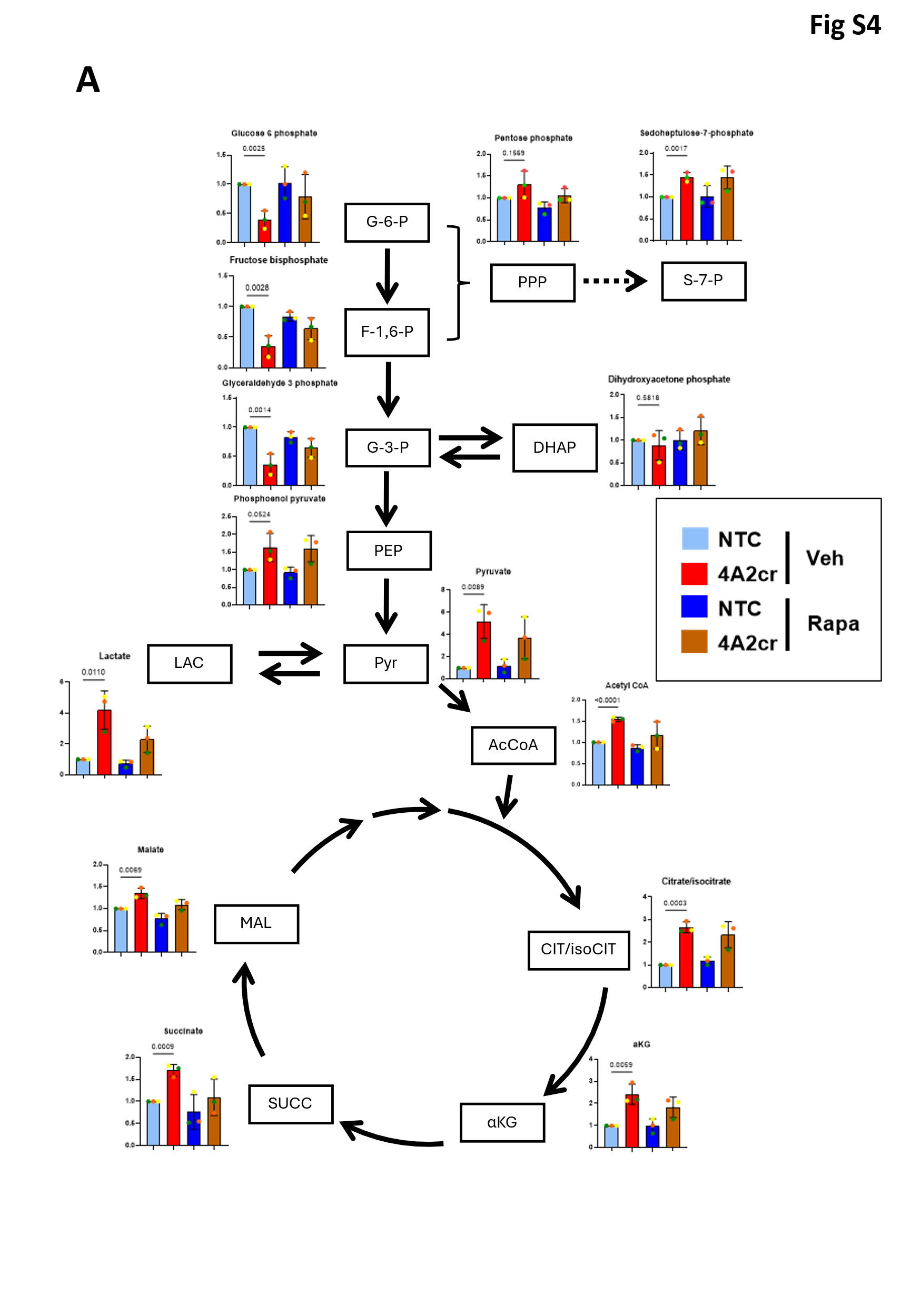


**Supplementary figure S4. eIF4A2 depletion influences metabolite levels**

(**A**) KM^NTC^ or KM^4A2cr^ cells were plated onto six-well dishes and allowed to adhere overnight. Adherent cells were incubated at 37°C for 24 hr in the presence and absence of rapamycin (1 μM) or vehicle control (Veh.). Cell lysates were used for extraction of polar metabolites. LC-MS was used to determine metabolites and levels of these are plotted as peak areas normalised to those of KMNTC cells. Bars are mean ± SEM, N=3, each coloured dot denotes an individual experiment. Statistical test is ANOVA with Tukey’s multiple comparison.


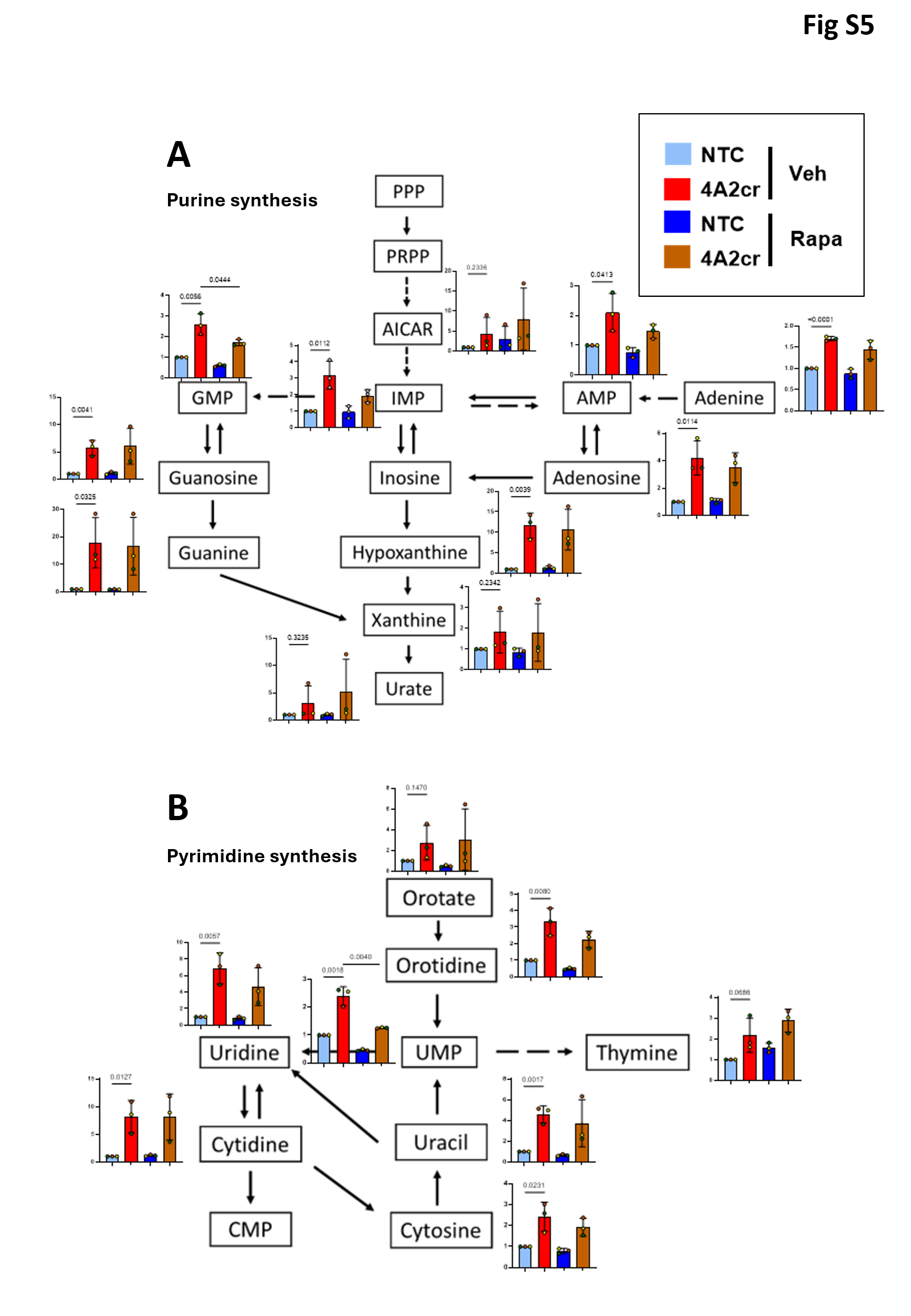


**Supplementary figure S5. eIF4A2 influences nucleotide synthesis and metabolism**.

KM^NTC^ or KM^4A2cr^ cells were plated onto six-well dishes and allowed to adhere overnight. Adherent cells were incubated at 37°C for 24 h in the presence and absence of rapamycin (1 μM) or vehicle control (Veh.) and polar metabolites were extracted after treatment. LC-MS was used to determine metabolites known to be involved in purine (A) and pyrimidine (B) synthesis and/or metabolism and levels of these are plotted as peak areas normalised to those of KM^NTC^ cells. Values are mean ± SEM, N=3, each coloured dot denotes an individual experiment. Statistical test is ANOVA with Tukey’s multiple comparison.


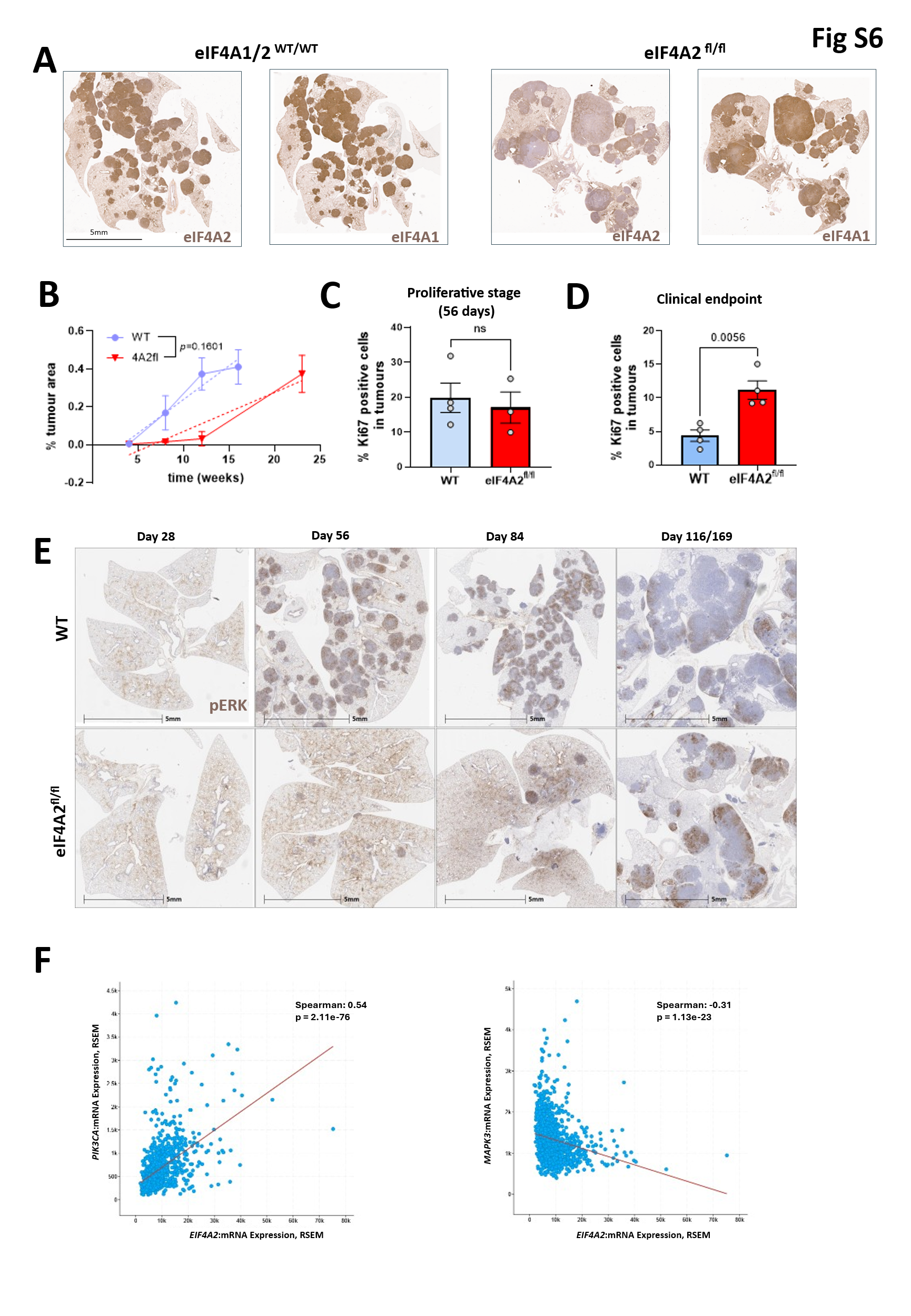


**Supplementary figure S6. Characteristics of eIF4A2 knockout tumours.**

(A-E) Ad5-SPC-CRE was administered intranasally to *Kras*^LSL-G12D/WT^; *Rosa26*^LSL-MYC/LSL-MYC^ (KM) mice that were *Eif4a2*^WT/WT^, or *Eif4a2*^fl/fl^. Mice were sacrificed at clinical endpoint and lungs removed and fixed and stained for eIF4A1, eIF4A2 (**A**) or phospho-ERK1/2 (**E**) using immunohistochemistry. In **(B)**, H&E was used to quantify tumour area overtime in KM-eIF4A2^WT/WT^ vs KM*-*eIF4A2^fl/fl^ mice and results were adjusted by linear regression. Each data point is the average of n=3-13 individual mice and *p-value* represents the difference in slope for each condition. For **(C)** and **(D)** mice were sacrificed at 56 days post induction or clinical endpoint and stained for the proliferative marker Ki67. Bars are mean + SEM of N=3-4 individual mice. Statistical test is t-test. (**F**) A NSCLC study (1053 patients) from Pan Cancer Atlas was queried in c-Bioportal for RNA expression of *EIF4A2*, *PI3K* and *ERK1 (MAPK3*). Graphs show Pearson and Spearman correlations of *EIF4A2* with either *PI3K* (positive correlation) or *ERK1* (negative correlation).


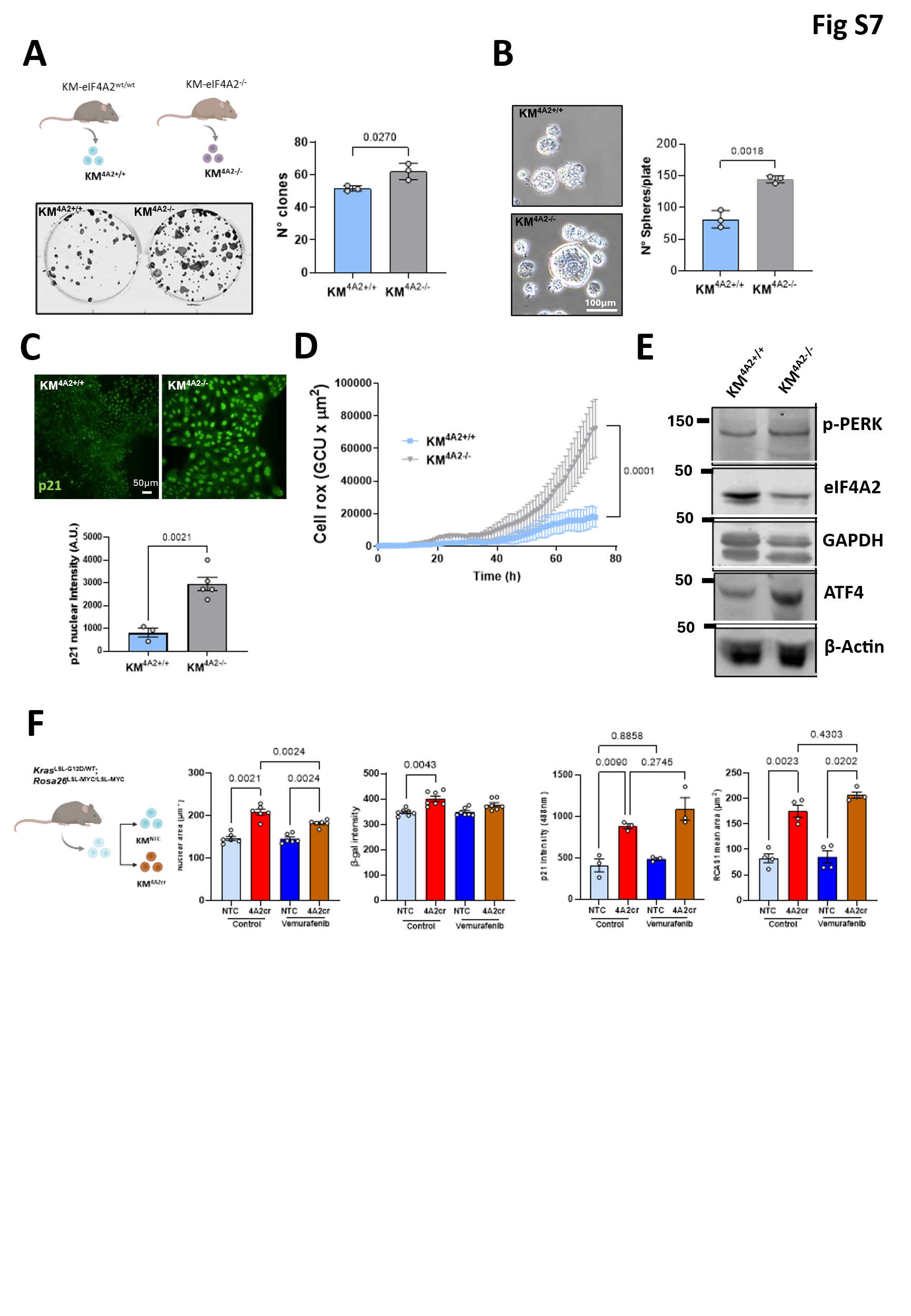


**Supplementary figure S7. Phenotypic analysis of KM^A2+/+^ and KM^A2-/-^ cells and Vemurafenib effects on senescence markers on KM^NTC^ and KM^4A2cr^ cells.**

(**A, B**) KM^A2+/+^ and KM^A2-/-^ cells were plated at low density onto plastic dishes in serum containing medium (**A**) or into low adhesion wells in serum-free medium (**B**). The resulting number of colonies (**A**) and tumour spheres (**B**) respectively were then determined. Bars are mean ± SEM, N=3 individual experiments, statistical test is unpaired t-test.

(**C, D**) KM^A2+/+^ or KM^A2-/-^ cells were plated onto plastic surfaces and allowed to grow for 48 hr. p21 expression was visualised and quantified using immunofluorescence (C) Bars are mean ± SEM N=3-5 individual experiments, statistical test is unpaired t-test. Cellular reactive oxygen species was determined using CellROX reagent (D). Data points are mean ± SEM N=3 individual experiments, statistical test is unpaired t-test.

(**E**) Activity of unfolded protein response signalling in KM^A2+/+^ and KM^A2-/-^ cells was assessed using western blotting with antibodies recognising phospho-PERK and ATF4. β-actin and GAPDH were used as loading controls.

(**F**) KM^NTC^ or KM^4A2cr^ cells were plated onto glass bottom plates and treated with 5μM Vemurafenib for 24h. Nuclear area and β-galactosidase activity, p21 expression and area of the Golgi ( RCAS1 is a cis-Golgi marker), were visualised and quantified using immunofluorescence. Graph bars are mean ± SEM of N=3-6 individual experiments, statistical test is one-way ANOVA.


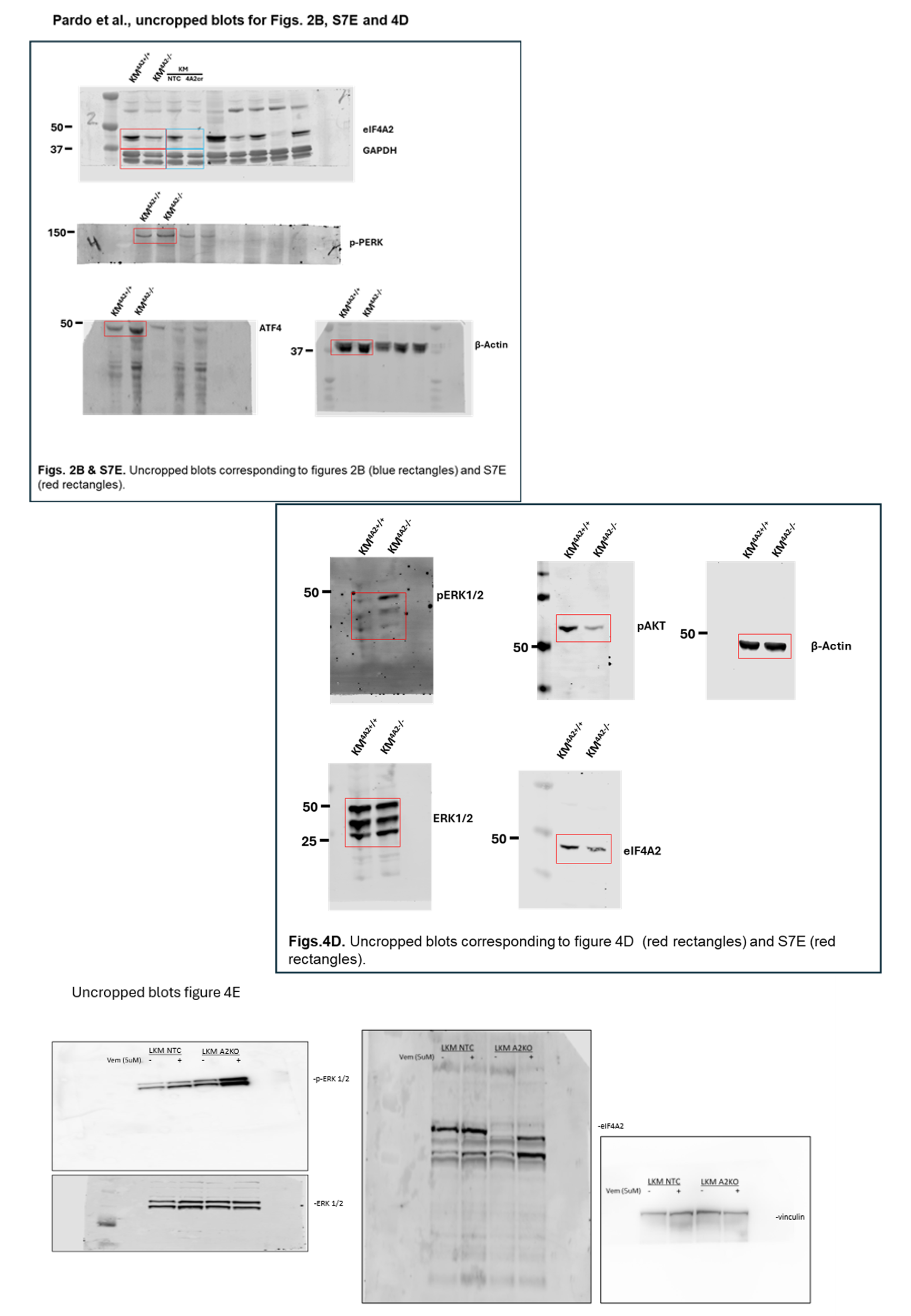

Supplement: Supplementary file 1 — Supplementary Material 1. [file 12943_2026_2680_MOESM1_ESM.docx]
